# Local-scale feedbacks influencing cold-water coral growth and subsequent reef formation

**DOI:** 10.1038/s41598-022-24711-7

**Published:** 2022-11-27

**Authors:** Guillem Corbera, Claudio Lo Iacono, Gonzalo Simarro, Jordi Grinyó, Stefano Ambroso, Veerle A. I. Huvenne, Furu Mienis, Marina Carreiro-Silva, Inês Martins, Beatriz Mano, Covadonga Orejas, Ann Larsson, Sebastian Hennige, Andrea Gori

**Affiliations:** 1 Institut de Ciències del Mar, CSIC, Barcelona, Spain; 2 University of Southampton, Southampton, UK; 3 National Oceanography Centre, Southampton, UK; 4 Royal Netherlands Institute for Sea Research (NIOZ), Texel, The Netherlands; 5 Institute of Marine Research-Okeanos, University of Azores, Ponta Delgada, Portugal; 6 Centro Oceanográfico de Gijón, Instituto Español de Oceanografía (IEO-CSIC), Avenida Príncipe de Asturias 70 Bis, 33212 Gijón, Spain; 7 Hanse-Wissenschaftskolleg – Institute for Advanced Study (HWK), Lehmkuhlenbusch 4, 27753 Delmenhorst, Germany; 8 University of Gothenburg, Gothenburg, Sweden; 9 School of GeoSciences, University of Edinburgh, Edinburgh, UK; 10 Università del Salento, Lecce, Italy; 11 Universitat de Barcelona, Barcelona, Spain

**Keywords:** Ecology, Ecophysiology, Ecosystem ecology

## Abstract

Despite cold-water coral (CWC) reefs being considered biodiversity hotspots, very little is known about the main processes driving their morphological development. Indeed, there is a considerable knowledge gap in quantitative experimental studies that help understand the interaction between reef morphology, near-bed hydrodynamics, coral growth, and (food) particle transport processes. In the present study, we performed a 2-month long flume experiment in which living coral nubbins were placed on a reef patch to determine the effect of a unidirectional flow on the growth and physiological condition of *Lophelia pertusa*. Measurements revealed how the presence of coral framework increased current speed and turbulence above the frontal part of the reef patch, while conditions immediately behind it were characterised by an almost stagnant flow and reduced turbulence. Owing to the higher current speeds that likely promoted a higher food encounter rate and intake of ions involved in the calcification process, the coral nubbins located on the upstream part of the reef presented a significantly enhanced average growth and a lower expression of stress-related enzymes than the downstream ones. Yet, further experiments would be needed to fully quantify how the variations in water hydrodynamics modify particle encounter and ion intake rates by coral nubbins located in different parts of a reef, and how such discrepancies may ultimately affect coral growth. Nonetheless, the results acquired here denote that a reef influenced by a unidirectional water flow would grow into the current: a pattern of reef development that coincides with that of actual coral reefs located in similar water flow settings. Ultimately, the results of this study suggest that at the local scale coral reef morphology has a direct effect on coral growth thus, indicating that the spatial patterns of living CWC colonies in reef patches are the result of spatial self-organisation.

## Introduction

The intricate structure of reef-building cold-water coral (hereafter referred as CWC) frameworks and the complex morphology of the thickets they form, promote an increase in the spatial heterogeneity of environmental conditions providing a wide range of habitats for a highly diverse and abundant associated fauna^1–3^. Consequently, CWC assemblages are increasingly known to be biodiversity and biomass hotspots comparable to their shallow and tropical counterparts^4–6^.

With the presence of favourable conditions, colonial scleractinian CWCs can grow and form three-dimensional complex reefs that entrap the by-passing hemipelagic sediments^7–9^, at some sites promoting the formation of coral mounds that can be over 300 m in height^10–12^. Unlike their warm-water counterparts, CWC reefs are commonly formed by a limited number of species, with the most well-known one being *Lophelia pertusa* (syn. *Desmophyllum pertusum*^13^). Although knowledge on the physiology and ecology of CWCs has considerably improved over the last decades^14–16^, the dynamic processes controlling reef growth and morphology in relation to environmental variables are still poorly understood^17^. Traditionally, the morphology of CWC reefs has been hypothesised to be driven by the underlying geological features (e.g., faults, scarps, ridges^11,12,18^) and by the hydrodynamics of the region (i.e., current direction and speed^19–22^). In regions where hard substrate is not limiting and bottom currents are mainly unidirectional, most coral reefs/mounds present a cigar- or teardrop-shaped morphology (e.g., Cape Lookout mounds, W Atlantic^23^; Florida mounds^24,25^; Norwegian mounds^26–28^). In such environmental settings, living coral is almost exclusively reported on the upstream side of the reef/mound structures, whereas the coral rubble on their tail is progressively older, indicating that CWC reefs/mounds grow into the flow^11,26,27,29^. In the strait of Florida, besides growing into the current, coral reefs also form semi-parallel bands that are orientated perpendicularly to the N–S dominant water flow^25^, suggesting that under certain environmental conditions CWCs might present a self-organising behaviour^30^.

CWCs tend to grow upwards into the water column seeking for water currents delivering food^31^. As the colonies develop into reefs, the coral framework starts to modify the local physical conditions and food availability^9,32,33^, ultimately initiating a series of positive and negative feedbacks that promote suitable and unsuitable environments for coral growth^30,34^. As a consequence, CWC reefs tend to display well-developed topographic highs with living colonies, alternated with low relief areas covered by coral rubble and fine sediments^31,35,36^. Although the interactions between extrinsic (i.e., water flow dynamics and food availability) and intrinsic factors (i.e., organism physiology) play an important role in the distribution and growth of coral colonies along a reef, and ultimately in its morphology, these are yet to be properly described and quantified.

As CWC reefs form over geological timescales^14,37,38^, it is challenging to elucidate and quantify the main environmental variables and feedbacks that regulate their geomorphological development. So far, studies that relate the variations in water flow dynamics caused by reef morphology with growth patterns of the reef itself are still scarce, and only based on numerical simulations^17,34^. Nevertheless, *L. pertusa* growth rates are fast enough (i.e., 0.01–38.1 mm year^–1^ linear extension) to perform relatively short-term growth experiments (i.e., 2–6 months^39–42^). In fact, many aquaria experimental studies have determined the presence of differential CWC growth rates under contrasting treatments of several environmental variables (e.g., temperature, pH and food supply) in relatively short periods of time^43–46^. According to experimental and numerical simulation studies, coral patches/reefs promote an alteration of the water flow and sediment transport in their vicinity^47^, enhancing particulate matter residence time and deposition, and ultimately affecting its availability within the reef itself^33,48^. Indeed, Mienis et al.^33^ demonstrated that, under realistic current speeds, reef patches promote higher flow velocities over the patch, whereas its wake is characterised by near-stagnant flows with reduced turbulence and vertical mixing. Similarly, water refreshment rates are drastically reduced, potentially affecting the oxygen exchange rates in and around the patch. However, all these studies do not take into account the response of living coral specimens. Therefore, there is a significant knowledge gap in quantitative experimental studies that help to understand how hydrodynamics and particle transport processes interact with reef/thicket morphology and affect coral growth, to ultimately regulate the geomorphological development of CWC reefs.

Based on previous studies^17,33^, we hypothesised that:

- Corals on the upstream side will grow faster (i.e. will accumulate more skeleton mass over the same time frame) than those on the downstream side.
- Corals on the downstream side will experience less suitable conditions than those on the upstream side, thus promoting a higher expression of stress-related enzymes.
- At a certain distance behind the reef, the water flow will be re-established to pre-reef conditions, allowing settled larvae to experience suitable living conditions and potentially give rise, with time, to the formation of a new coral reef patch.

## Results

### Hydrodynamic features

**Figure 1:**
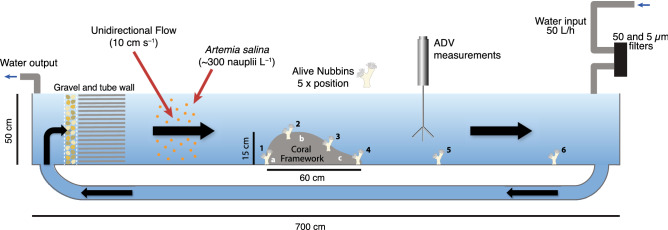
Schematic representation of the flume experimental setting, were the water was continuously renewed (50 L h^–1^). A gravel and tube wall were placed 50 cm from the flume start to homogenise the flow. An asymmetric artificial reef structure (15 cm × 60 cm × 90 cm), with the highest point at x = 20–30 cm, was located 2.25 m from the flume start with the first 4 sets of nubbins located along the reef: at the front (x_1_ = 0 cm), top (x_2_ = 15 cm) and back (x_3_ = 45 cm and x_4_ = 55 cm). Lastly, two more nubbin sets were placed x_5_ = 1 m and x_6_ = 3 m behind the reef structure. White letters refer to the different panels in Fig. 3.

**Figure 2:**
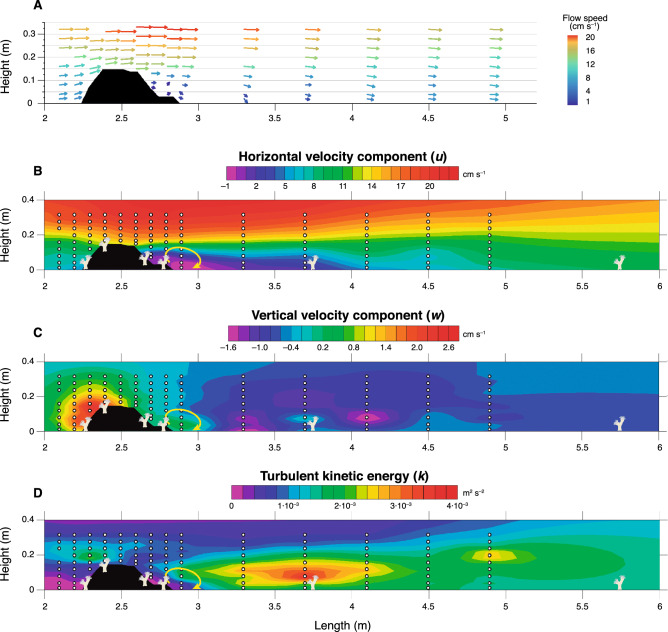
Vector plots of horizontal (*u*) and vertical (*w*) average flow speed along the flume and over the coral reef structure (**A**). Heat plots of the hydrodynamic measurements acquired with an ADV, including the horizontal (*u*) and vertical (*w*) velocity components (**B**, **C**) and turbulent kinetic energy (*k*; **D**). The yellow arrow in plots (**B**–**D**) indicates the presence of a vertical eddy behind the reef structure.

**Figure 3:**
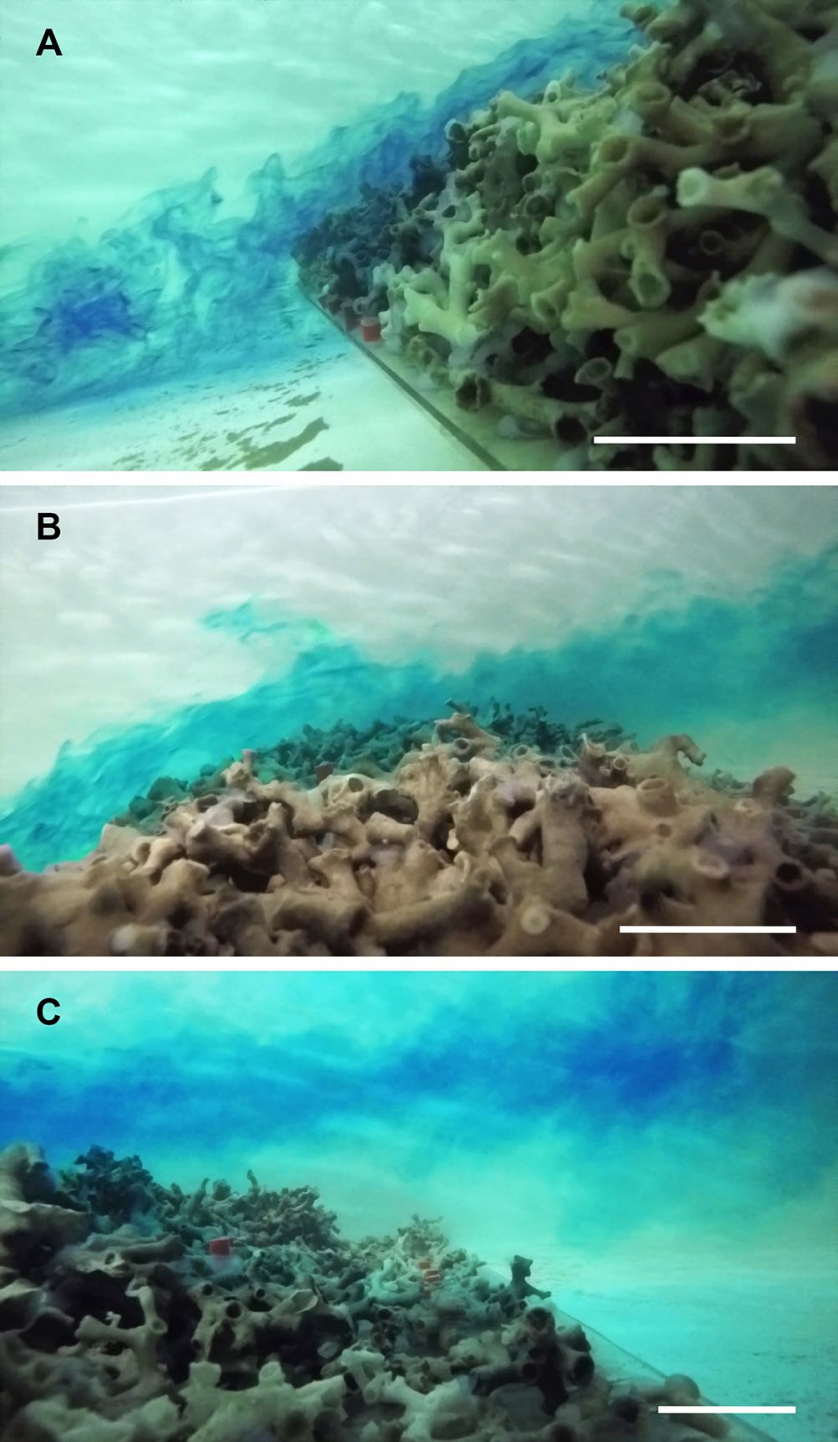
Dye trials showing the flow deflection caused by the coral framework at the upstream (**A**), top (**B**) and downstream (**C**) sides of the reef, portraying how the zooplankton-rich waters would avoid the downstream nubbins. The white lines are displayed for scale and correspond to ~ 5 cm.

Flow speed heat plots of *u* (horizontal component) and *w* (vertical component) showed considerable variations between upstream and downstream sides of the reef patch, which occupied the whole width of the flume, extended for a length of 60 cm and had a maximum height of 15 cm (Figs. 1, 2A,B). Before reaching the reef patch, *u* average velocities (i.e. dominant component of velocity) ranged from 7 to 18 cm s^–1^ at the near-bottom and water surface, respectively, thus exposing the first set of coral nubbins to an average horizontal velocity of ~ 7–8 cm s^–1^ (Fig. 2A). While passing over the reef patch, *u* velocities increased to an average value of ~ 14 cm s^–1^ at the top-frontal sector of the reef, where the second set of coral nubbins was located (Fig. 2A). Above the reef patch, the water velocity augmented up to 20 cm s^–1^. This accelerated water layer was still observable ~ 1 m behind the 60 cm long reef patch. In contrast, immediately downstream of the reef patch and below its maximum height (z < 15 cm), *u* velocities were reduced and became almost stagnant, with values between – 1 and 4 cm s^–1^ (Fig. 2A). As a consequence, the third and fourth sets of coral nubbins were exposed to horizontal velocities of < 2 cm s^–1^, being shadowed by the reef patch itself from the nauppli-rich waters (Fig. 3C). Horizontal velocities did not recover the pre-reef hydrodynamic conditions until 1.5 m behind the reef patch, which resulted in the fifth set of coral nubbins being subjected to an average *u* velocity of ~ 4 cm s^–1^ (Fig. 2A). Lastly, the sixth set of coral nubbins, located almost 3 m behind the reef patch, was exposed to an average velocity of ~ 8–9 cm s^–1^.

Pre-reef vertical velocities (*w*) were nearly 0, only to become slightly positive (i.e. flowing upwards at < 3 cm s^–1^) at the frontal part of the reef patch, coinciding with the first two sets of coral nubbins (Fig. 2B). At the wake of the reef, *w* values gradually shifted to turn slightly negative (i.e. flowing downwards at ~ 0.8–2 cm s^–1^) from ~ 0.35 to ~ 2 m behind the structure (Fig. 2B). Indeed, the horizontal and vertical velocity heat plots reveal the presence of an eddy immediately behind the reef patch (Fig. 2A,B). Along the whole flume length, *w* velocities at > 0.3 m from the bottom were nearly 0, thus indicating that the water surface was unaffected by the presence of the reef structure.

Similar to the flow speed heat plots, turbulent kinetic energy (*k*) values also displayed considerable variations between upstream and downstream regions (Fig. 2C). The first set of coral nubbins, located at the frontal and lower part of the reef structure (*z* = 0 m), was exposed to k values close to 0. A first peak of *k* = 2.7·10^–3^ m^2^ s^–2^ was observed above the frontal part of the reef (*x* = 2.3 m, *z* = 0.16–0.24 m), coinciding with the second set of coral nubbins (Fig. 2C). In contrast, the third and fourth set of nubbins, located at the leeside of the reef, were subjected to practically no turbulent activity. Turbulence increased again behind the reef patch from the flume bottom up to 20 cm into the water column, with values of up to 3.9·10^–3^ m^2^ s^–2^ occurring below the reef’s maximum height and between 0.45 and 1.25 m behind it. The *k* maxima coincided with the location of the fifth set of coral nubbins, located 0.85 m behind the reef (Fig. 2C). Beyond this region, turbulent activity was kept higher than in pre-reef regions until the end of the flume.

Overall, hydrodynamic measurements revealed increased *u*, *w* velocities and turbulence above the frontal part of the reef patch, while its leeside was characterised by slow flowing vertical eddies and negligible turbulence. Further behind the reef, negative vertical velocities and increased turbulence indicate vertical mixing, with maximum values occurring almost 1 m behind the reef.

### Coral growth

**Figure 4:**
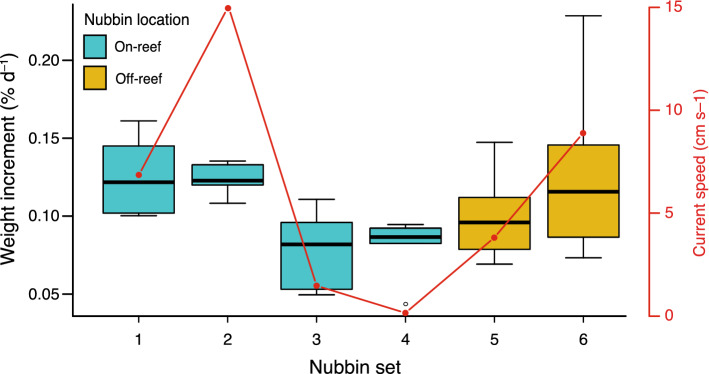
Average percental weight increment of each coral nubbin set (1–6), together with the average current speed measured in each location.

Coral growth rates for each nubbin set are expressed as average percentual increment in coral weight per day, together with the standard deviation. For the whole set of corals used in the experiment, growth rates ranged between 0.043 and 0.23% d^–1^, with a mean value of 0.11 ± 0.04% d^–1^. The first two sets of nubbins, located at the lower-frontal and upper-frontal part of the reef structure, displayed growth rates ranging from 0.10 to 0.16% d^–1^ with average values of 0.13 ± 0.03% d^–1^ and 0.12 ± 0.01% d^–1^, correspondingly (Fig. 4). At the leeside of the reef, the third and fourth sets of coral nubbins showed growth rates ranging from 0.04 to 0.10% d^–1^, with an average value of 0.08 ± 0.03% d^–1^ and 0.08 ± 0.02% d^–1^, respectively (Fig. 4). Lastly, the fifth and sixth sets of nubbins, located 1 and 3 m behind the reef structure, presented growth rates that ranged between 0.07 and 0.22% d^–1^, with mean values of 0.10 ± 0.03% d^–1^ and 0.13 ± 0.06% d^–1^ (Fig. 4).

Coinciding with the variations observed in the hydrodynamic patterns between upstream and downstream sectors, statistically significant differences (Kruskal–Wallis test; p < 0.05) in growth rates were observed among the coral nubbin sets (Fig. 4). Nonetheless, after performing pairwise comparisons using a Wilcoxon Rank Sum test, significant differences (p < 0.05) were only observed between the 1st and 2nd coral nubbin sets, located at the frontal part of the reef, and the 3rd and 4th, located at its leeside (Figs. 1, 4).

### Protein expression

**Figure 5:**
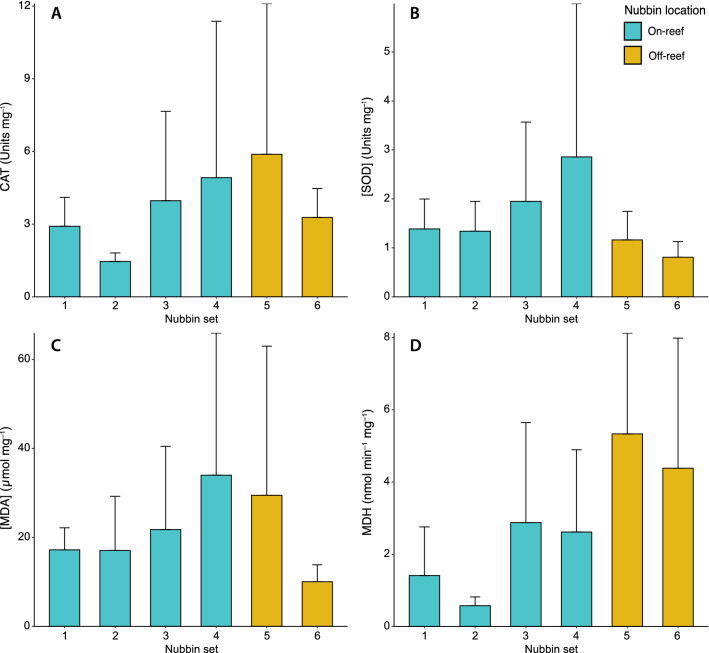
Specific mean activity (Units per mg of total protein) values and standard deviation of (**A**) Superoxide dismutase (SOD), (**B**) Catalase (CAT), (**C**) MDA (lipid peroxidation subproduct) mean concentration (µmol per mg of total protein) and (**D**) malate dehydrogenase (MDH).

Catalase (CAT) and Superoxide dismutase (SOD) are known to be general antioxidant alarm indicators, with higher activities of these enzymes representing greater oxidative alarms in the organism^49^. The coral nubbins used in this experiment displayed overall CAT activity values ranging from 0.86 to 16.74 U mg^–1^ of protein, with a mean value of 3.74 ± 3.93 U mg^–1^ of protein. The first two sets of nubbins showed activities ranging between 0.98 and 4.61 U mg^–1^ of protein, with average activities of 2.91 ± 1.19 and 1.46 ± 0.35 U mg^–1^ of protein, respectively (Fig. 5A). The third and fourth coral nubbin sets located at the leeside of the reef showed higher CAT activity values, ranging from 0.86 to 16.40 U mg^–1^ of protein, which resulted in mean activity values of 3.97 ± 3.69 and 4.92 ± 6.46 U mg^–1^ of protein, correspondingly (Fig. 5A). In contrast, the fifth and sixth sets of nubbins showed CAT activities with values ranging between 1.61 and 16.74 U mg^–1^ of protein and mean activity values of 5.88 ± 6.22 and 3.28 ± 1.19 U mg^–1^ of protein (Fig. 5A). The overall SOD activity values ranged from 0.49 to 8.33 U mg^–1^ of protein, with a mean value of 1.58 ± 1.53 U mg^–1^. The two sets of nubbins located at the frontal part of the reef patch presented activity values that ranged from 0.73 to 2.31 U mg^–1^ of protein and average values of 1.38 ± 0.61 and 1.34 ± 0.61 U mg^–1^ of protein (Fig. 5B). The highest activity values of SOD were observed in the two nubbin sets located at the leeside of the reef, which presented values ranging between 0.52 and 8.33 U mg^–1^ of protein, and mean activity values of 1.95 ± 1.62 and 2.86 ± 3.13 U mg^–1^ of protein (Fig. 5B). The two sets of off-reef nubbins displayed the lowest activity ratios with values varying from 0.49 to 1.74 U mg^–1^ of protein and mean activity values of 1.16 ± 0.58 and 0.81 ± 0.32 U mg^–1^ of protein (Fig. 5B).

Lipid Peroxidation (LPO), defined by the malondialdehyde (MDA) concentration, indicates the level of cell damage^50^. The whole set of coral nubbins presented MDA concentrations between 5.13 and 88.75 µmol mg^–1^, with an average concentration of 21.59 ± 20.89 µmol mg^–1^. The two coral nubbin sets located at the frontal part of the reef patch and the third set of nubbins showed intermediate MDA concentrations, ranging from 9.04 to 53.28 µmol mg^–1^. The average values for these three sets were 17.21 ± 4.95, 17.05 ± 12.19 and 21.78 ± 18.68 µmol mg^–1^, respectively (Fig. 5C). In contrast, the fourth and fifth nubbin sets presented the highest average LPO values, with concentrations that ranged from 5.13 to 88.75 µmol mg^–1^ and average values of 33.99 ± 32.94 and 29.47 ± 33.51 µmol mg^–1^ (Fig. 5C). The lowest average values of LPO were observed in the sixth set of nubbins, which displayed a range of 5.15–14.12 µmol mg^–1^ and a mean concentration of 10.04 ± 3.81 µmol mg^–1^ (Fig. 5C).

Besides being involved in the Krebs cycle, Malate dehydrogenase (MDH) also has an antioxidant role in preventing oxidative stress damage through the action of oxaloacetate^51^. Hence, when combined with other stress-related enzymes, it can provide information on the physiological condition of the coral nubbins. The activity values of the nubbins used in this experiment ranged between 0.29 and 9.45, with an average activity of 3.28 ± 2.89 nmol min^–1^ mg^–1^. For this enzyme, the first two nubbin sets showed the lowest activity ratios, varying from 0.29 to 7.31 nmol min^–1^ mg^–1^ and with mean activities of 1.41 ± 1.35 and 2.26 ± 3.37 nmol min^–1^ mg^–1^ (Fig. 5D). The third and fourth nubbin sets displayed values ranging between 0.46 and 6.89 nmol min^–1^ mg^–1^, and showed mean activities of 2.87 ± 2.77 and 2.62 ± 2.28 nmol min^–1^ mg^–1^ (Fig. 5D). The last two nubbin sets showed the highest activity ratios, ranging from 0.41 to 9.45 nmol min^–1^ mg^–1^, with average values of 5.33 ± 2.79 and 4.38 ± 3.59 nmol min^–1^ mg^–1^ (Fig. 5D).

Despite the data presented for stress-related protein activity and/or concentration seems to show that the coral nubbins from sets 3–5 are in a worse physiological condition, no statistically significant differences (p > 0.05) were observed.

## Discussion

By using living *L. pertusa* nubbins and flow conditions similar to those occurring in actual CWC reef settings, the present flume experiment provides novel empirical data on the effects of geomorphologically steered water flow distortions on coral growth, physiological condition and its subsequent influence on reef formation along a scaled artificial CWC reef patch.

In terms of hydrodynamics, the results acquired here match with those of Mienis et al.^33^, which demonstrates the reproducibility and robustness of the experiment. For similar pre-patch *u* velocities (10–15 cm s^–1^), the coral patch/reef used in the present study (15 cm tall) promoted variations to the hydrodynamics of the flume similar to those caused by 10 and 25 cm tall coral patches in Mienis et al.^33^, with *u* velocities increasing up to 21 cm s^–1^ above the reef and near-stagnant water observed at the seafloor just behind the coral structure. This region of reduced *u* velocities could be observed up to 2 m behind the reef both in the present study and in Mienis et al.^33^. In terms of turbulence, a high *k* region was observed, in the present experiment, above the upstream part of the reef patch with values of up to 2.7·10^–3^ m^2^ s^–2^, which likely promote enhanced mixing within the benthic boundary layer, thus replenishing the reef with particles^52^ and increasing their encounter rates with the coral nubbins. With regards to the downstream off-reef region, the most turbulent area was observed at a distance between ~ 0.4 and 2 m behind the reef patch and from the flume bottom up to 0.2 m into the water column. Nonetheless, the *k* values measured here (i.e. 1.4–3.9 × 10^–3^ m^2^ s^–2^) were considerably higher than those observed in Mienis et al.^33^, for both the 10 and 25 cm tall patches (i.e. ~ 0.4–2 × 10^–3^ m^2^ s^–2^).

**Figure 6:**
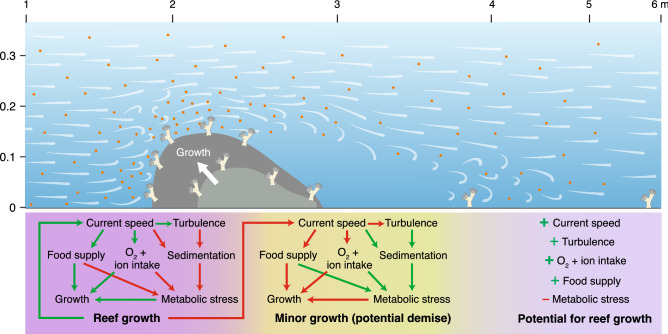
Schematic representation of the expected reef growth, together with the positive and negative feedbacks occurring on the upstream and downstream sides of the reef structure. Green and red arrows indicate the presence of positive and negative feedbacks respectively. Orange dots represent *Artemia salina* nauplii. Initial nubbin location corresponds to that of the present experiment.

The buoyant weight results acquired confirm our first hypothesis, demonstrating that the nubbins located on the upstream part of the reef patch, exposed to high water velocities (i.e. 7–15 cm s^–1^) and low to moderate turbulence, displayed a significantly higher skeletal growth than those subjected to a near-stagnant water flow at the leeside of the reef (i.e. 0.4–1.7 cm s^–1^; Figs. 2, 4). If the growth rates acquired in this study (i.e. 0.043–0.23% d^–1^) are compared, by means of the conversion factors presented by Büscher et al.^42^, to in situ linear extension rates measured in corals from environmentally contrasting regions (i.e. Gulf of Mexico, Mediterranean Sea, North Sea; 2.4–35 mm year^–1^ ≈ 0.046–0.21% d^–1^)^53–55^, it can be observed that our results cover the whole growth rate range of *L. pertusa* colonies. Hence, on top of being statistically significant, the variations in growth rates among nubbin sets also seem to be biologically relevant. Indeed, the first two sets of nubbins likely benefited, from the higher turbulence occurring above the reef front and their direct exposure to the incoming nauplii-rich waters. In addition, high flow speeds considerably enhance particle encounter rate and thus, the intense currents going through the first two nubbin sets might have helped increasing their zooplankton capture rates^56,57^. The fast growth of these nubbins contrasts with the results of Purser et al.^58^, where the authors observed that *L. pertusa* displayed higher zooplankton capture rates under reduced flow speeds (i.e. < 5 cm s^–1^). However, the latter experiment consisted of small coral nubbins with a reduced potential to modify water hydrodynamics, whereas the larger reef patch used here affected water flow direction and speed, ultimately benefiting the upstream nubbins. In addition, Orejas et al.^59^ witnessed that there were no significant differences in terms of polyp expansion for colonies exposed to flow speeds ranging from 0.5 to 15 cm s^–1^, which combined with the growth rates acquired here, suggests that flow speeds > 5 cm s^–1^ might still be suitable for the feeding of *L. pertusa*. Indeed, long-term in situ measurements from coral reefs show average flow speeds of 10 cm s^–1^, albeit with generally high variability in terms of flow direction and velocity along time^60,61^. This was also discussed by Duineveld et al.^60^, as in situ video observations from the Mingulay Reef showed that at current speeds of ~ 40 cm s^–1^ the percentage of extended polyps was at its maximum. Besides the role current velocity plays in food supply, it also affects the thickness of the diffusive boundary layer (DBL), which is the region of water closest to the corals, where molecular diffusion dominates the transport between the water column and the organism^62–64^. Higher current velocities generally reduce the thickness of the DBL, thus enhancing the rate of diffusion of inorganic nutrients and dissolved gasses between the water column and coral tissues^63–66^. In this sense, the corals located on the upstream side of the reef were exposed to higher current velocities (Fig. 2A), likely reducing the thickness of their DBL and potentially enhancing the intake of Ca^2+^, CO_3_^2−^ and HCO_3_^–^, all of which are important to coral calcification^63,67^ (Fig. 6). Hence, the potentially higher particle encounter rate and enhanced diffusion of ions involved in the calcification process might have been enough to stimulate coral growth in the upstream side of the reef patch (Fig. 6). Although particle encounter and ion diffusion rates were not actually measured here, the former hypothesis is further supported by the low activity values and concentration of stress-related enzymes (i.e. CAT, SOD, MDA and MDH) in the first two nubbin sets (Fig. 5), which denote a good physiological condition. Nonetheless, future research efforts should be directed towards acquiring a better understanding on the actual role of particle encounter/capture rates and ion intake rates in promoting coral growth.

In contrast, the nubbins at the downstream side of the reef were shadowed by the coral framework itself, which deflected the fast flowing and nauplii-rich waters over it (Figs. 2, 3), likely decreasing the food supply (Fig. 6). Despite this was not measured, even if some zooplankton were present in this part of the reef, the particle encounter rate of the coral nubbins would potentially still be very low due to the extremely reduced flow speeds (i.e. < 2 cm s^–1^), which promote particle deposition. Although several studies have observed that *L. pertusa* colonies may survive under suboptimal feeding conditions using their energetic reserves over a period of months^15,68,69^, if this scenario is maintained for longer periods, it can potentially cause the demise of the coral colonies due to starvation^17^. In addition to the low food conditions, the increased residence time at the wake of the reef patch might have reduced the oxygen renewal of the water surrounding the nubbins. This combined with a thicker DBL, caused by the low water velocity^63–66^, likely reduced the oxygen, Ca^2+^, CO_3_^2−^ and HCO_3_^–^ intake of the coral nubbins. Indeed, the slower growth rates of the downstream nubbins coincided with a higher activity of antioxidant enzymes (CAT and SOD), a greater cellular damage (MDA) and an increased demand for MDH activity to compensate stress^51^, which suggest these corals were in worse physiological conditions than the upstream ones (Fig. 5). Although the results are not statistically significant, the differential concentration and activity of stress-related enzymes suggest that as expected, the downstream nubbins are subjected to unsuitable environmental conditions that affect their development and physiological condition. Along with the decreased food supply, oxygenation and ion intake, the hydrodynamics measured in this experiment also show the presence of a vertical eddy just behind the reef structure (Fig. 2). If this feature is combined with the almost stagnant water flow and extrapolated to naturally occurring reefs, it would result in enhanced sediment deposition at the leeside of the reef^33,48,70^ (Fig. 6). This is a detrimental situation for coral growth, as the corals have to invest their limited energy in removing sediment particles through mucus production and/or ciliary action, which over long periods can be fatal for *L. pertusa*, as the polyps may become energetically depleted^71^.

As the reef grows towards the current, it would establish a positive feedback at the reef front involving intense hydrodynamics that would promote a potential increase in oxygenation, food supply and ion intake, ultimately endorsing coral growth. Conversely, the reef back would be subjected to a negative feedback of almost stagnant water promoting a potentially lower degree of oxygenation and ion intake, enhanced sedimentation, decreased food supply and increased metabolic stress, possibly causing coral demise (Fig. 6). This would promote a cigar- or teardrop-shaped reef morphology that would coincide with that of naturally occurring reefs exposed to unidirectional water flows, such as the ones found in the Florida strait^24,25^, Cape Lookout^23^ and off the Norwegian coast^26–28^. Indeed, these reefs can be divided in three contrasting regions that match with our results (i.e. living colonies growing on the upstream part of the reef followed by blocks of dead coral framework and a final zone of coral rubble)^31^. Both the data acquired here and in situ observations^23–27^ contrast with the results of Hennige et al.^17^, where a simulated colony subjected to a unidirectional water flow developed into an hemispherical coral patch, with living coral on its outer rim and dead coral framework in the inner parts of the patch. However, it is important to note that the theoretical results in Hennige et al.^17^ do not consider the distribution of particulate organic matter or 3-Dimensional flow in the experimental setting, which are affected by the varying size and morphology of the growing patch, and as demonstrated in this study are two critical variables regulating reef development.

With regards to the off-reef downstream region, the higher turbulence observed there likely enhanced vertical mixing^52^, potentially providing the 5th and 6th nubbin sets with a higher availability of food particles than those located at the immediate downstream side of the reef patch (i.e. sets 3 and 4). Indeed, when comparing the growth rates of downstream reef nubbins and off-reef ones (i.e. sets 5 and 6), a tendency towards greater skeletal growth was observed in the latter, albeit with no statistically significant differences (Fig. 4). This trend also corresponds with the water flow gradually recovering pre-reef velocities towards the end of the flume (Fig. 2A), allowing for a higher oxygenation and ion intake. Although the 5th set of nubbins were located in an area with enhanced vertical mixing (Figs. 2C, 6), an important proportion of the nauplii might still have flown over them due to the flow deflection caused by the reef. If this is considered, and although these nubbins were subjected to slightly higher water velocities (i.e. ~ 3.5–4.5 cm s^–1^) than the downstream ones (i.e. sets 3 and 4), the environmental conditions were likely not sufficient to provide a suitable particle encounter rate that would promote a differential coral growth. Besides the potentially low food supply that the 5th set of nubbins received, the high turbulence to which they were subjected to might have also been a factor of stress. Indeed, the 5th nubbin set displayed some of the highest activities and concentrations of enzymes related with oxidative stress, cellular damage and energy expenditure (Fig. 5).

In contrast, the 6th set of nubbins was subjected to a flow speed similar to those experienced by the upstream ones (i.e. ~ 9 cm s^–1^), albeit with higher turbulence (Fig. 2). Such current velocity together with its downward direction, likely promoted and enhanced oxygen and ion intake, as well as a sufficient food supply to stimulate coral growth. Although the average coral growth of the 6th nubbin set showed a similar value to that of the upstream ones (i.e. sets 1 and 2; Fig. 4), a higher internal variability of growth rates was detected. This in turn, resulted in no significant differences being observed between the 6th nubbin set and the downstream ones (i.e. sets 3 and 4). Similarly, if the activities and concentrations of enzymes related to oxidative alarm response and cellular damage are considered, only the 6th set of nubbins presented comparable values to those of the upstream reef ones, except for the MDH activities (Fig. 5). The high MDH activity values of the 6th nubbin set suggest that these corals are using additional energy to compensate some kind of physiological stress (e.g. turbulence)^66^. This constant energy investment might have left some of the corals with less energy to be invested on skeletal growth, thus promoting a higher variability in their growth rates. A further potential explanation for the absence of significant differences in terms of coral growth between the downstream nubbin sets and the 6th set, could be the limited duration of the experiment (~ 2 months). Indeed, we hypothesize that if the experiment had lasted longer (4–6 months), more significant differences in terms of coral growth and stress-related enzyme expression would have been observed between the downstream nubbins (i.e. sets 3 and 4) and the set of nubbins located 3 m behind the reef (i.e. set 6). While the results acquired here are promising, they are not entirely conclusive and they should be interpreted with caution.

Although our third hypothesis cannot be confirmed, the hydrodynamic, growth rate and physiological data acquired for the off-reef nubbins suggest that at some distance behind the reef, the environmental setting could become favourable enough to promote the rise of a potential new coral patch (Fig. 6). If this pattern was kept at a regional scale, it could promote the formation of a series of spatially generated reefs, such as the ones present off the coasts of Florida and Norway^25,28^. In cases where coral recruitment is linked to individual dropstones, the patterns observed here would lead to the formation of several cigar- or teardrop-shaped reefs, whereas in more homogenous hard substrate conditions it could promote the development of lines of reef growing perpendicularly to the main flow, whereby the distance between lines is determined by reef height and width^25^. Indeed, Bøe et al.^72^ described that the ridge-like coral mounds in the Hola Through are 20 m high in average and they are generally distributed parallel to each other, ~ 300 m apart, following the main direction of the current (i.e. northerly or northwesterly). Although at different scales, these results are in accordance with our experiment, as hydrodynamic data and coral growth measurements suggest that with a reef of 15 cm in heigh, suitable conditions for the formation of a potential new coral patch may occur at a distance of ~ 250–350 cm downstream of the reef (15–20 times the reefs’ height). Nonetheless, this distance is probably dependent on bottom current speeds, since mounds influenced by stronger currents would be expected to cast larger water flow shadows. Lastly, if all the feedbacks are considered, faster growing reefs will also expand the area influenced by near-stagnant waters and decreased food supply, hampering the development of other reefs located directly in their wake. These underdeveloped reefs could eventually be absorbed by the growing ones or buried under layers of sediments^18,73–75^.

The present study provides empirical data that reveal the presence of local-scale positive and negative feedbacks for coral reef growth, mediated by the framework geomorphology itself. This suggests that spatial patterns of living CWC colonies in reef patches are the result of spatial self-organisation. Nonetheless, the specific role of all the factors driving these patterns is yet to be properly described. Therefore, future studies should focus on the variables that might be affected by the interaction between reef morphology and hydrodynamics (e.g. suspended particle distribution, ion intake), to better understand live coral distribution on growing reefs and its potential implications for reef/mound formation over long time spans.

## Conclusions

- Similar to what is naturally observed in unidirectional current settings, the results acquired in this experiment confirm that a potential colony/reef would slowly grow into the current, whereas coral colonies or polyps from the same colony living on the leeside of the coral structure would most likely die due to the absence of a sufficient food supply.
- Local-scale positive and negative feedbacks regulated by the reef geomorphology itself occur in different parts of the reef, even affecting the physiological condition of living coral nubbins.
- The water flow reestablishment and food particle distribution several meters behind the reef structure (≥ 3 m) could allow the formation of a new reef patch, thus giving rise to a potential spatial pattern of successive parallel reefs developing in a cyclic manner.
- The results obtained here suggest that scleractinian CWC reefs exhibit spatial self-organisation, which might help towards better understanding living coral distribution on reefs, as well as reef morphology and distribution along the seafloor. However, the changes in food supply and ion intake rates related to morphology-derived hydrodynamic variations, that potentially promote such self-organisation, are yet to be properly quantified. Hence, future experiments should be directed towards improving our knowledge on the actual role of these two factors on coral growth.

## Material and methods

### Coral collection, maintenance and preparation

Living fragments of *L. pertusa*, approximately 10–30 cm in size, were collected by means of the ROV Max-Rover (Hellenic Centre of Marine Research, Greece) from four different locations of the Cabliers Coral Mound Province (Alboran Sea, Southwestern Mediterranean) and during the SHAKE cruise, conducted on board the R/V Sarmiento de Gamboa in April–May 2015. Collection from the Coral Mound Province was at ~ 300 m water depth in the frame of the IRIS EU FP7 Eurofleets2 project (EF2-EE-003). Coral fragments were kept alive on board and then transferred to the Institut de Ciències del Mar (ICM-CSIC) in Barcelona (Spain), where they were maintained in a 200 L water tank. The water was kept at ~ 12 °C and continuously renewed with Mediterranean Sea water pumped from 15 m depth and filtered at 50 μm (Polypropylene Net Filter)^76^. A submersible pump (Sicce Nano 2000) with a flow rate of 2000 L h^−1^ provided continuous water movement within the tank. While in the maintenance tank, corals were fed 5 times per week with frozen *Mysis* and *Artemia salina* nauplii. Prior to the start of the experiment, 30 coral nubbins containing between 3 and 5 polyps were cut from the original colonies and mounted on labelled bases using epoxy resin.

### Experimental setting

The experiment was conducted in a 3150 L flume (7 m long × 0.9 m wide × 0.5 m high) equipped with a motor-controlled propeller with a variable rotational speed (Fig. 1). A wall of pebbles (3–5 cm) and a plastic-tube wall (15 cm long pvc tubes) oriented along the current main direction were placed at 50 cm from the flume’s beginning to homogenise the flow (Fig. 1). An Acoustic Doppler Velocimeter (ADV-Vectrino) was used to calibrate the propeller rotational speed and fix the flow speed at 10 cm s^−1^ for the entire duration of the experiment, which was chosen according to the average in situ flow speed observed in thriving CWC habitats^77,78^. Flume water was continuously renewed with Mediterranean seawater pumped from 15 m depth at a rate of 120 L h^–1^ and filtered by 5 µm (Polypropylene Net Filter). Constant water temperature of ~ 12 °C was maintained with two chillers (Teco TK 2000).

Dead *L. pertusa* fragments were cleaned with a 10% H_2_O_2_ solution to remove organic matter and used to build a coral reef patch on a methacrylate panel. The base of the reef structure was built using small coral fragments (< 5 cm long), collected from coral rubble deposits of CWC reefs. Larger coral branches (up to 25 cm long) were assembled together (glued with silicone) on top of the base until the desired reef patch dimensions and shape were achieved. Size and shape of the coral structure were scaled to real reef/mound dimensions^19,36^. The reef occupied the whole width of the flume (i.e., y = 90 cm), had a length of x = 60 cm, and a maximum height of z = 15 cm at x = 20–30 cm (Fig. 1). The height was limited to 15 cm in order to leave space enough to simulate an infinite water depth on top of the reef patch, avoiding a significant footprint of the coral framework on the water surface elevation. The coral structure was placed at 2.25 m from the flume start (Fig. 1), where measured freestream conditions were stable. The living coral nubbins were placed at 6 different positions along the flume. Four of these were within the coral reef structure, whereas the other two were located 1 and 3 m behind the structure (Fig. 1). Within the coral reef structure, nubbins were placed on its frontal part, on its top (15 cm from the start of the structure), and at its rear side (45 and 55 cm from the start of the reef structure; Fig. 1). Five coral nubbins, that worked as replicates, were located at each of the described locations along the flume.

During the experiment, coral nubbins were fed once a day with freshly hatched *A. salina* nauplii, with a concentration of ~ 300 nauplii L^−1^ within the flume. Water temperature was monitored every 5 min by means of a HOBO Pro V2 logger, which registered a mean temperature of 12.3 ± 0.4 °C during the experiment. Nutrient concentrations were measured every week and showed values below the average values for north-western Mediterranean shallow waters^79^ (i.e. < 200 m water depth), with a mean NO_3_^−^ + NO_2_^−^ concentration of 4.8 ± 1.4 µmol l^–1^, NH_4_^+^ of 2.26 ± 0.41 µmol l^–1^, SiO_4_^2−^ of 1.69 ± 0.61 µmol l^−1^ and PO_4_^3−^ of 0.19 ± 0.04 µmol l^−1^. Particulate organic carbon (POC) was measured once a week before and after feeding the corals by filtering 2 l of flume water through a GFF (0.7 µm) glass fibre filter (Whatman, 47 mm diameter). Mean POC concentration was 0.10 ± 0.02 mg C l^−1^ before, and 0.29 ± 0.12 mg C l^−1^ just after feeding the corals.

### Hydrodynamic measurements

Water flow measurements were acquired by means of the ADV at three different stages of the experiment: (1) in freestream conditions prior to the placement of the coral reef structure, (2) after the placement of the reef structure but before placing the living coral nubbins in the flume, and (3) at the end of the experiment, after removing the nubbins from the flume. All water flow measurements were performed without any living corals in the flume to previously investigate which were the best locations to place the reef structure and the nubbin sets, in order to properly test our hypotheses. Before the coral reef structure was placed in the flume, 8 vertical profiles with measurements at 2, 4, 8, 16 and 32 cm above the flume bed were carried out every 50 cm along the flume. Based on these measurements, the position of the coral reef structure within the flume was established at 2.25 m from the flume start, where the flow showed a well-developed boundary layer. This also left sufficient space behind the reef structure for the water flow to potentially recover pre-reef conditions. The second set of measurements, taken at a higher horizontal resolution on and behind the reef structure, allowed to assess in detail how the horizontal velocity component of the current (*u*) was modified by the interaction between the reef structure and the downstream flow. This allowed to define the locations of the living coral nubbins within the reef structure and along the flume, in order to expose them to contrasting current regimes and turbulence conditions. The above-mentioned vertical profiles, were carried out every 10 cm over the coral reef structure and every 40 cm behind it. Finally, the last set of measurements, performed at the end of the experiment, consisted of vertical profiles collected from 2.0 to 4.9 m along the flume length. The profiles were separated by 10 cm from 2.0 to 2.9 and by 40 cm beyond that point. In order to obtain highly detailed profiles of horizontal (*u*) and vertical (*w*) velocity components together with turbulence kinetic energy (*k*), the number of vertical measurements was increased to acquire data at 2, 4, 8, 12, 16, 20, 24, 28 and 32 cm above the flume bed (Fig. 2). For each sampling point, 1000 measurements were acquired over 5 s. Time averaged horizontal (*u*) and vertical (*w*) components were calculated by computing the average of all measurements obtained for each sampling point. Outliers that indicated unreliable data (values > 2σ) were filtered and removed from the measurements. Turbulent kinetic energy (*k*) was calculated in order to determine the amount of vertical mixing occurring along the flume, which has an effect on the food distribution across the water column. Data of downstream velocity (*u*), vertical velocity (*w*) and turbulence (*k*) were gridded using the software SURFER 11 by means of a kriging method with a linear variogram model. Within the linear variogram model, anisotropy ratio was set to 4 in order to give more importance to horizontal data similarities and avoid the creation of artefacts on the plot. Dye trials were also performed at the end of the experiment to observe how the reef deflected the flow and affected food distribution in the water column behind it (Fig. 3).

### Coral growth measurements

The weight of the 30 living coral nubbins used in the experiment was measured at the start (6 of March, 2018) and end (27th of April, 2018) of the experiment by means of the buoyant weight method^62,80^, using a Mettler AT 261 analytical balance with a precision of 0.1 mg. This technique allows for the determination of the skeletal dry weight of the coral nubbins from their weight in seawater^80^. In order to take the weight of the bases and the epoxy resin into account, the nubbins were weighted before and after mounting them on the bases. Immediately after finishing the experiment, all coral nubbins were flash-frozen, conserved at − 80 °C, and subsequently sent to the Instituto do Mar (IMAR) in Azores (Portugal), for protein expression analyses. Coral nubbin growth was expressed in percentage of weight per day^40^. Normality of the data and variance homogeneity were tested by means of a Shapiro–Wilks and Bartlett tests, prior to the analyses. As the data did not follow a normal distribution and neither displayed variance homogeneity, growth rates of the coral nubbins from different positions along the flume were compared by means of a non-parametric Kruskal–Wallis test. A Pairwise Wilcoxon Rank Sum test was used to determine which sets of coral nubbins presented differences. All statistical analyses were carried out by means of the statistical software R 3.2.0^81^.

### Protein expression

#### Preparation of tissue extracts and protein quantification

Coral nubbins were crushed in a mortar and pestle with liquid nitrogen to extract 1 g of sample. All samples were homogenized at a 1:5 w/v ratio in an ice-cold 10 mM phosphate-buffered saline solution (PBS pH 7.4) containing 5 mM EDTA and 1% (v/v) ProteaseArrest™ (G-Biosciences®) cocktail. An Ultra Turrax (Ystral®, D79282) was used to slowly increase the rotational velocity from 8000 to 20,000 rpm during the ~ 2 min extraction time. Enzyme activities were measured in the supernatant fraction after the homogenate was centrifuged at 16,000×*g* and 4 °C for 30 min. All enzyme assays were tested with commercial enzymes obtained from Sigma®. Each sample was run in triplicate (technical replicates). Total soluble proteins content was quantified according to Bradford method^82^, adapted from Bio-Rad Bradford microassay set up in a 96-well microplate. Absorbance was read at 595 nm in a microplate reader (Thermo Scientific™). A calibration curve was created using bovine serum albumin (BSA; Bio-Rad) standards (0–1 mg ml^−1^).

#### Determination of antioxidant enzyme activities and lipid peroxidation

Total superoxide dismutase (SOD) activity was characterised spectrophotometrically through an indirect method^83^ based on the competition of SOD with 2-(4-iodophenyl)-3-(4-nitrophenol)-5-phenyltetrazolium chloride (I.N.T) for dismutation of the superoxide anion (O^2−^). In this method, O^2−^ radicals that quantitatively react with I.N.T to form a red formazan dye were generated by means of xanthine and xanthine oxidase. Absorbance was measured at 505 nm and 25 °C, 30 s after the addition of xanthine oxidase as start reagent across a 180 s incubation period (Ransod SD 125kit, Randox). One unit of SOD is defined as the amount of enzyme needed to inhibit the rate of formazan dye formation by 50%. Subsequently, a SOD standard curve was employed to correlate percent inhibition of samples with SOD activity. The following equation was used to calculate the percent inhibition of standards and samples: 100 − [[ΔA505/min]/[ΔAblank/min]] × 100. The overall SOD activity in all tissues is expressed as Units per milligram of protein (U mg^−1^) in the sample wet weight.

Catalase (CAT) activity was measured spectrophotometrically by measuring the rate of H_2_O_2_ disappearance at 240 nm (extinction coefficient, ε = 0.04 mM^−1^ cm^−1^) and 25 °C during a 180 s incubation period^84^. A total reaction volume of 2.7 ml was obtained through the combination of 50 mM potassium phosphate buffer (pH 7.0) and 13.5 mM H_2_O_2_ as a substrate, initiated by the addition of sample into quartz cuvettes with a path length of 10 mm. In order to validate the assay, catalase from bovine liver (Sigma®) was used as a positive control (1524 U ml^−1^). The following equation was used to calculate Catalase activity: [ΔA240/min/0.04] × [total volume/sample volume]. CAT enzymatic activity in all tissues is expressed as Units per milligram of protein (nmol min^−1^ mg^−1^) in the sample wet weight.

Lipid peroxidation (LPO) values were acquired through the quantification of a specific end-product (malondialdehyde, MDA) of the oxidative degradation process of lipids^85^. A colorimetric reaction which uses 1-methyl-2-phenylindole (MPI) as chromogen (Randox Ltd.) was used to measure concentrations of MDA. A chromophore with an absorbance maximum at 586 nm is generated from the condensation of one molecule of MDA with 2 molecules of MPI under acidic conditions. Concentrations of MDA in tissue samples were calculated using a standard curve prepared with freshly prepared solutions of malondialdehyde bis [dimethyl acetal] (ACROS Organics™) and values were displayed as nmol mg^−1^ of total protein in the sample wet weight.

The activity of the Malate dehydrogenase (MDH) was performed according to protocol. Nubbins samples were homogenized at a 1:5 w/v ratio in ice-cold 50 mM imidazole buffer (pH 7.4) containing 5 mM EDTA and 1% (v/v) ProteaseArrestTM (G-Biosciences®) and centrifuged (10,000×*g*, 4 °C for 30 min). Activity was measured by adding 2 µl of supernatant to 1 ml of the reaction mixture containing 50 mM imidazole buffer (pH 7.4), 0.5 mM oxaloacetate, and 0.15 mM NADH at room temperature. The enzyme activity was measured by the decrease in absorbance at 340 nm, and was expressed as nmol NAD(P)^+^ formed per milligram of tissue protein per minute.

Normality of the data and variance homogeneity were tested by means of a Shapiro-Wilks and Bartlett tests, prior to the statistical analyses. Since the data did not follow a normal distribution nor displayed homogeneity of variance, the activity and/or concentration of stress-related proteins in coral nubbins from different positions along the flume were compared by means of a non-parametric Kruskal–Wallis test.

## Data Availability

Data available from the Zenodo Digital Repository: https://doi.org/10.5281/zenodo.6773359.
